# Mg-supplementation attenuated lipogenic and oxidative/nitrosative gene expression caused by Combination Antiretroviral Therapy (cART) in HIV-1-transgenic rats

**DOI:** 10.1371/journal.pone.0210107

**Published:** 2019-01-22

**Authors:** Lama ElZohary, William B. Weglicki, Joanna J. Chmielinska, Jay H. Kramer, I. Tong Mak

**Affiliations:** Department of Biochemistry and Molecular Medicine, The George Washington University, Schoold of Medicine and Health Sciences, Washington, D.C., United States of America; Max Delbruck Centrum fur Molekulare Medizin Berlin Buch, GERMANY

## Abstract

We determined if HIV-1 expression in transgenic (HIV-1-Tg) rats enhanced hepatic genomic changes related to oxidative/nitrosative stress and lipogenesis during cART-treatment, and assessed effects of Mg-supplementation. A clinically used cART (atazanavir-ritonavir+Truvada) was given orally to control and HIV-1-Tg rats (18 weeks) with normal or 6-fold dietary-Mg. Oxidative/nitrosative and lipogenic genes were determined by real-time RT-PCR. cART induced a 4-fold upregulation of sterol regulatory element-binding protein-1 (SREBP-1) in HIV-1-Tg-rats, but not in controls; Tg rats displayed a 2.5-fold higher expression. Both were completely prevented by Mg-supplementation. Nrf2 (Nuclear erythroid-derived factor 2), a master transcription factor controlling redox homeostasis, was down-regulated 50% in HIV-Tg rats, and reduced further to 25% in Tg+cART-rats. Two downstream antioxidant genes, heme oxygenase-1(HmOX1) and Glutathione-S-transferase(GST), were elevated in HIV-Tg alone but were suppressed by cART treatment. Decreased Nrf2 in Tg±cART were normalized by Mg-supplementation along with the reversal of altered HmOX1 and GST expression. Concomitantly, iNOS (inducible nitric oxide synthase) was upregulated 2-fold in Tg+cART rats, which was reversed by Mg-supplementation. In parallel, cART-treatment led to substantial increases in plasma 8-isoprostane, nitrotyrosine, and RBC-GSSG (oxidized glutathione) levels in HIV-1-Tg rats; all indices of oxidative/nitrosative stress were suppressed by Mg-supplementation. Both plasma triglyceride and cholesterol levels were elevated in Tg+cART rats, but were lowered by Mg-supplementation. Thus, the synergistic effects of cART and HIV-1 expression on lipogenic and oxidative/nitrosative effects were revealed at the genomic and biochemical levels. Down-regulation of Nrf2 in the Tg+cART rats suggested their antioxidant response was severely compromised; these abnormal metabolic and oxidative stress effects were effectively attenuated by Mg-supplementation at the genomic level.

## Introduction

Acquired immunodeficiency syndrome (AIDS) caused by HIV-1 was first formally recognized in patients in the USA in 1981 [1]. HIV disease continues to be a serious health issue for parts of the world [2]; worldwide, an estimated 37 million people are still living with the virus [3]. Antiretroviral therapy (ART), or HAART including nucleosides and non-nucleoside reverse transcriptase inhibitors (NRTI, NNRTI), integrase inhibitors and protease inhibitors (PI) ([4]) have been used to treat HIV infection for nearly two decades. With the introduction of combination anti-retroviral therapy (cART) consisting of 2 nucleoside analog inhibitors (NRTIs) plus 2 protease inhibitors (PIs), HIV-1 replication in infected patients was dramatically reduced to the extent that HIV-1 infection has become a more manageable disease [4,5]. However, along with the chronic use of NRTI—and PI-containing cART, significant side effects of oxidative/nitrosative stress, hyperlipidemia, and lipodystrophy occurred [6]; these side effects might contribute to the increased cardiovascular disease associated with chronic use of cART in HIV-1 patients [6,7]. Nevertheless, the role of HIV-1 infection/gene expression in the potential heightened susceptibility to cART-induced metabolic toxicity and systemic oxidative stress remains unclear. In a recent concurrent study [8], by using an established HIV-1 transgenic (Tg) rat model we found that a clinically used cART, consisting of Truvada (2 NRTIs) plus atazanavir-ritonavir (2 PIs), induced early oxidative stress resulting in cardiac dysfunction. In the present study, we focused at the molecular level, on key transcriptome changes related to lipogenesis and antioxidant/nitrosative responses.

Magnesium (Mg) is known to have direct anti- free radical and anti-calcium influx properties [9–12]. Mg-supplementation at high doses has been reported to provide clinical beneficial effects for various cardiovascular disorders such as hypertension, atherosclerosis and CAD [13–16]. By using normal control rats, we also reported the protective effects of Mg-supplementation against AZT and RTV-induced oxidative, endothelial and cardiac toxicity [17–19]. It is unclear whether these antioxidant and anti-calcium properties of Mg influenced cART-induced metabolic and related side effects in HIV-1 expressed Tg animals; more importantly, we examined whether any of the Mg protective effects were related to transcriptome modification.

## Materials and methods

### Animals and chemicals

Male 5 week-old Hsd:HIV-1 (F344) transgenic rats and the background wild type control (Fischer 344/NHsd) rats were obtained from Envigo/Harlan Laboratory (Indianapolis, IN) as described [8]. cART components (atazanavir-ritonavir plus Truvada) were obtained from The GWU-Pharmacy. The primers for the real-time quantitative PCR were obtained from BioSynthesis, Inc (Lewisville, TX).

All animal experiments were guided by the principles for the care and use of laboratory animals as recommended by the US Department of Health and Human Services and approved by The George Washington University (GWU) Animal Care and Use Committee [8]. A description of the Animal Research Facility (ARF) is online at our GWU ARF website: http://research.gwu.edu/office-animal-research. Following 1 week quarantine, all rats were maintained under aseptic conditions in individual sterilized hepa-filtered isolator cages in a dedicated room by GWU ARF. Rats were initially placed on an *ad libitum* irradiated chow (until they were 3 months old) and sterilized water, *and* were on a 12 h light/dark cycle.

At 3 months old, the control and HIV-1 Tg rats were divided into 8 groups: (A1) Control normal Mg group; (A2) Control + cART; (A3) Tg alone; (A4) Tg + cART (normal Mg); (B1) Control + high Mg; (B2) Control + cART+ high Mg; (B3) Tg + high Mg; and (B4) Tg + cART+ high Mg. Groups A1-A4 were fed a Mg-normal (0.1% MgO/kg food) diet and groups B1-B4 received a Mg-supplemented (0.6% MgO/kg food) diet. Both Mg diets, which contained extracted casein as the diet base and essential vitamins and nutrients, were irradiated and obtained from Envigo/Teklad (Madison, WI). The human fixed dosages of tenofovir [disoproxil fumarate] (TDF)/emtricitabine (FTC) in Truvada are 300 mg/200 mg/day which is equivalent to about 5.0/3.3 mg/kg/day; and for atazanavir (ATV)/ritonavir (RTV), the 300 mg/100 mg/day human dose is equivalent to about 5.0/1.65 mg/kg/day. Due to the higher (>6-fold higher) drug metabolic rates in rodents [20], the nominal dosage of the cART was designed to be about 3-fold higher than that of human on a per body weight basis. The levels of the drugs required to be mixed in the diet (performed by Teklad) were estimated based on the known average daily food consumption data provided by Teklad; the quantities of the drug components added per kg diet were: 350 mg TDF, 230 mg FTC, 350 mg ATV, and 115 mg RTV. The exact final daily dosages were calculated by the average daily amounts of diet consumed and were monitored on a weekly basis. The concurrent cART treatment on normal Mg diet, or high Mg diet was carried out for 18 weeks. At the end of the experimental period, all 8 groups of rats consumed comparable amounts of food (43–46 gm/kg/day) [8]; the averages of each group were within 6% of each other. Therefore, the final dosages of the cART components for both the control or Tg rats on normal or high Mg diets were estimated to be: atazanavir/ritonavir = 16.5/5.5 mg/kg/day; and for Truvada = 16.5 mg TDF plus 11 mg FTC/kg/day.

### Blood and liver samples

At sacrifice, blood samples were collected in sodium citrate containing tubes by cardiac puncture during euthanasia from non-fasting animals. Plasma samples were obtained following centrifugation at 250 g x10 min and stored at -80°C until needed. Liver samples were rapidly excised, processed, frozen, and stored at -80°C.

### Real time RT-PCR for selected genes

The expression of selected hepatic lipogenic gene plus several selected genes related to oxidative/nitrosative stress and inflammatory activity were determined by a real-time quantitative PCR procedure similar to that described [18,19]. Briefly, 30 mg of liver samples from each rat were homogenized extracted for total RNA by TRIzol reagent (Invitrogen) and further purified using RNeasy mini spin columns (Qiagen, Valencia, CA), according to the manufacturer’s procedures. cDNA was synthesized and amplified from total RNA using the iScript cDNA synthesis kit (Bio-Rad, Hercules, CA), and real-time quantitative PCR was performed using SsoAdvanced Universal SYBR Green Super Mix (BioRad) with the Applied Biosystems 7300 real-time PCR system. The hepatic expression of selected inflammatory/oxidative/nitrosative gene levels of TNFα, Nrf2, HmOX-1, GST, iNOS, and the key lipogenic transcription factor, SREBP-1 were determined and quantified with values normalized by 18s rRNA using forward: 5’GGATCCATTGGAGGGCAAGT3’ and reverse: 5’ACGAGCTTTTTAACTGCAGCAA3’ primers.

For the selected genes, the following primers were used:

SREBP-1, forward: 5’TCACAGATCCAGCAGGTCCCC3’ and reverse: 5’GGTCCCTCCACTCACCAGGGT3’;

Nrf2, forward: 5’CACATCCAGACAGACACCAGT3’ and reverse: 5’CTACAAATGGGAATGTCTCTGC3’;

HmOx-1, forward: AAGAGGCTAAGACCGCCTTC and reverse: GCATAAATTCCCACTGCCAC;

GST, forward: 5’CACAAGATCACCCAGAGCAA3’ and reverse: CCATAGCCTGGTTCTCCAAA;

iNOS, forward: 5’CACCTTGGAGTTCACCCAGT3’ and reverse: 5’TGTTGTAGCGCTGTGTGTCA3’.

TNFα forward: 5’TCAGCCTCTTCTCATTCCTGC3’ and reverse: 5’GTGCAGCATCGTTTGGTGGTT3’;

### Plasma total triglyceride, total cholesterol, and oxidative/nitrosative indices (8-isoprostane, 3-nitrotyrosine and RBC glutathione)

The plasma total triglyceride (TG) content was determined quantitatively by a colorimetric (at 570 nm) Triglyceride Assay kit (Cayman Chemical, Ann Arbor, MI) according to instructions. Plasma cholesterol levels were determined by a Cholesterol Fluorometric Assay kit (Cayman Chemical), and total cholesterol content for each sample was estimated on the basis of relative fluorescence (excitation 550 nm, emission 590) obtained from standards. Plasma 8-isoprostane levels at 18 weeks were determined by a Cayman Enzyme Immunoassay Kit as described [8, 17–19]. RBC reduced (GSH) and oxidized (GSSG) glutathione levels were assessed enzymatically by the DTNB-GSSG reductase method [17–19]. Plasma 3-nitrotyrosine (3-NT) results, determined by the enzyme immunoassay components from Cayman Chemical, were derived from the concurrent study just published [8].

### Statistical analysis

Data were checked by *F*-test for equality of groups’ variation and paired Student t-test for each subgroup versus reference subgroup. In general, data were expressed as means of 4–5 rats ± SEM (standard error of the mean). Significance was considered at p<0.05.

## Results

### Expression of the key hepatic gene related to lipid metabolism-sterol regulatory element-binding transcription factor-1 (SREBP-1)

**Fig 1:**
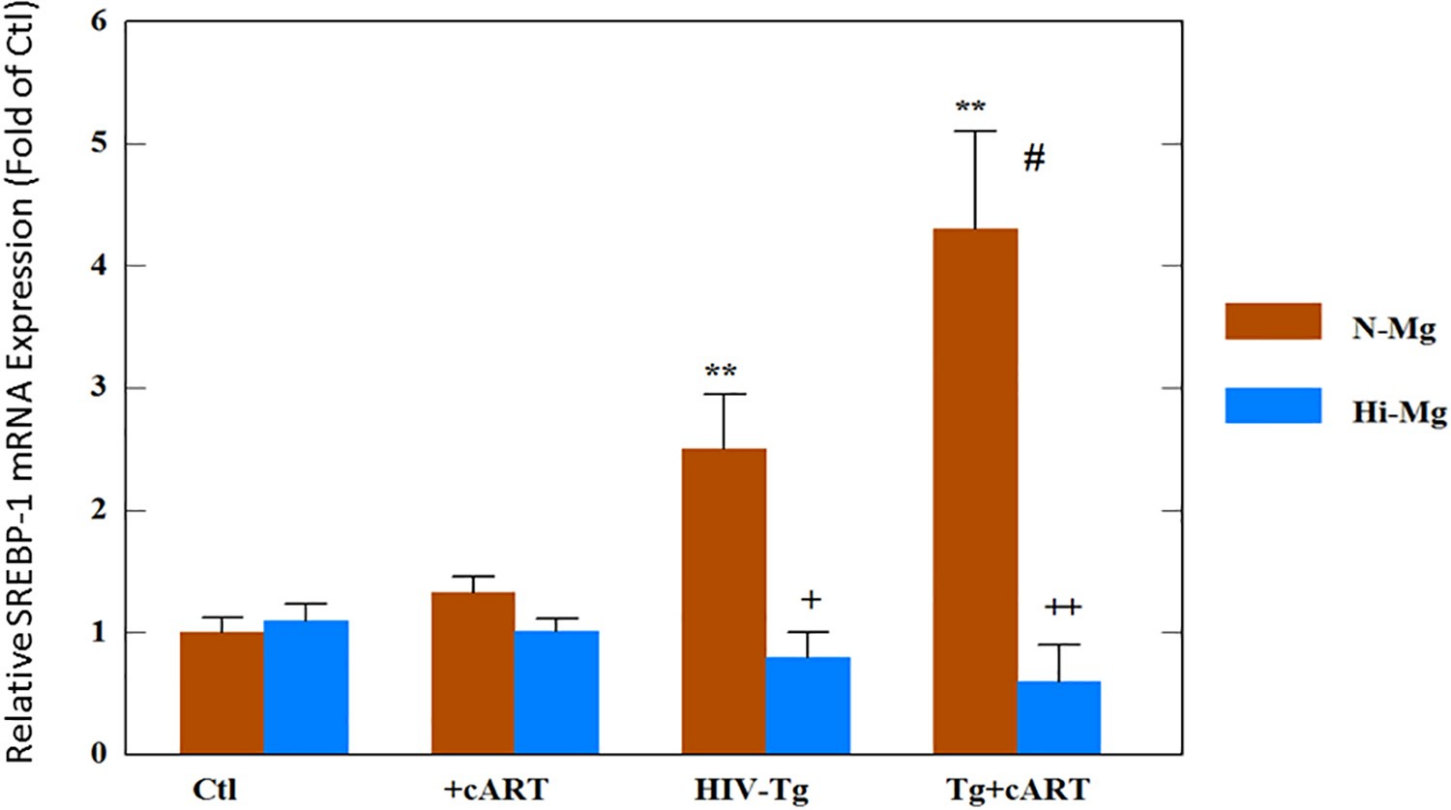
HIV-1 expression plus cART promoted hepatic expression of sterol regulatory element-binding protein-1 (SREBP-1)—Suppression by high Mg diet. Control or HIV-Tg rats received cART (atazanavir-ritonavir plus Truvada) for 18 months while on normal or high Mg (6-fold) diets. Real-time quantitative-PCR results were normalized by 18s rRNA, and the values are expressed as means of 4–5± SE; **p<0.01 vs. Ctl, + p<0.05, # p<0.05 vs. Tg alone, ++p<0.01 vs. corresponding normal Mg (N-Mg) groups.

**Fig 2:**
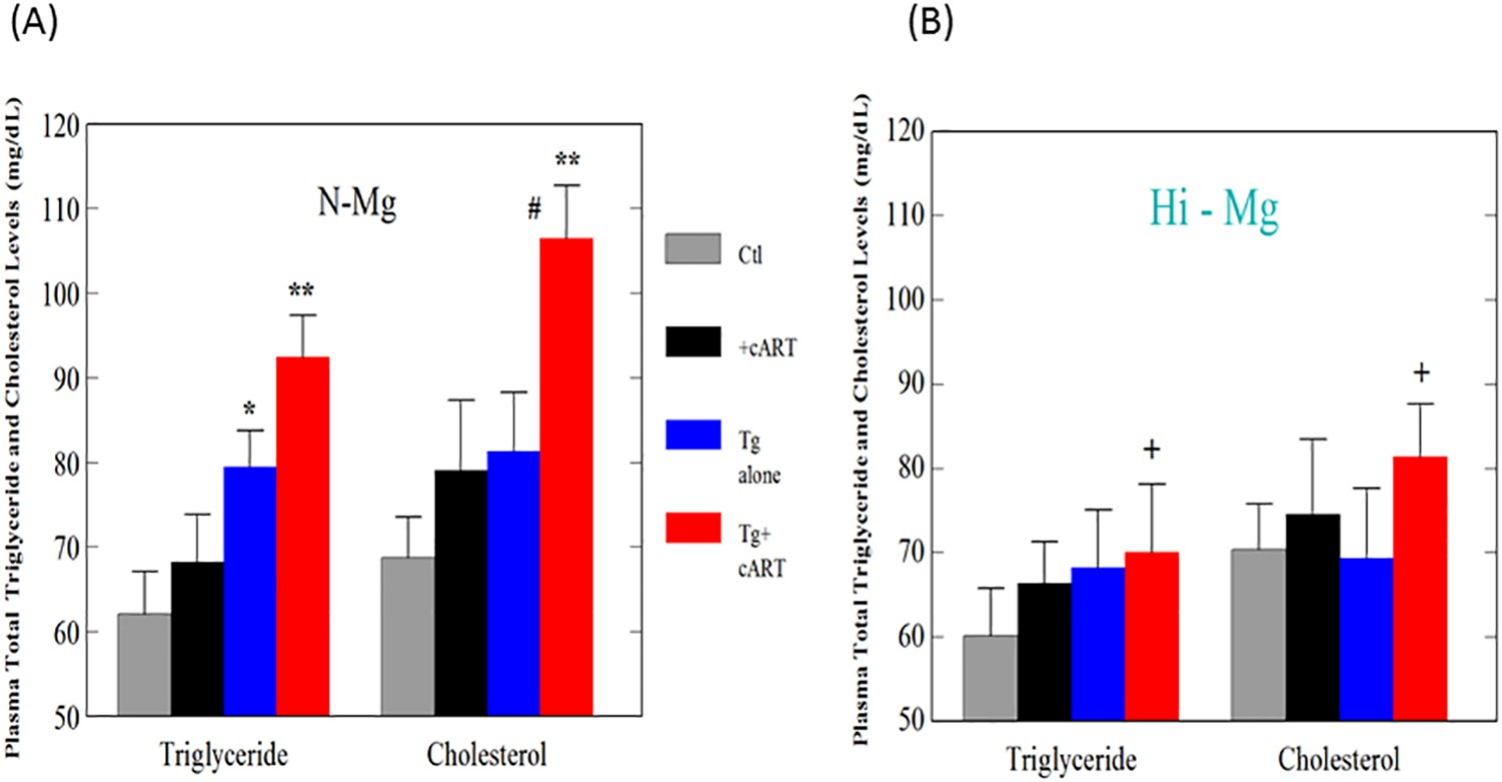
cART raised plasma total triglyceride and total cholesterol levels in HIV-Tg rats—Attenuation by high Mg diet. Control or HIV-Tg rats received cART for 18 months while on normal or high Mg diets; plasma total triglyceride (TG) or cholesterol levels were determined by Caymen assay kits (see Methods). Results are means of 5±SE; *p<0.05, **p<0.01 vs. Ctl, # p<0.05 vs. Tg alone, +p<0.05 vs. corresponding N-Mg groups.

The effects of cART, HIV-1 expression, and high Mg diet on the changes in the mRNA level of SREBP-1 were determined. As shown in Fig 1, HIV-Tg alone revealed about a 2.3-fold higher expression compared to the control; when treated with cART, the upregulation was further elevated to >4-fold higher in the transgenic rats (p<0.05 vs. Tg alone). It was noteworthy that the cART treatment did not induce any increase in the control rats (Fig 1). With Mg supplementation, upregulation due to either Tg alone or Tg + cART was completely reversed to levels slightly lower than the controls (Fig 1). To determine the correlations between SREBP-1 upregulation and potential hyperlipidemic effects, plasma cholesterol and triglyceride levels were also determined. The results showed that the triglyceride level was significantly elevated without treatment in the transgenic HIV rats (Fig 2A), and that the drugs only caused a small but non-significant increase in control rats. However, cART treatment of the HIV Tg rats induced the highest level of total triglyceride (Fig 2A). Likewise, the cholesterol level in the transgenic HIV Tg rats receiving cART was markedly and significantly elevated (Fig 2A). In parallel with its effects on SREBP-1 expression, Mg supplementation substantially and significantly suppressed both triglyceride and cholesterol elevations, especially in the cART- treated Tg rats (Fig 2B).

### Effects of cART, HIV-1 expression and high Mg on selected oxidative stress related genes: Nrf2 and the downstream antioxident genes, heme oxygenase-1 (HmOx-1) and glutathione S-transferase (GST)

**Fig 3:**
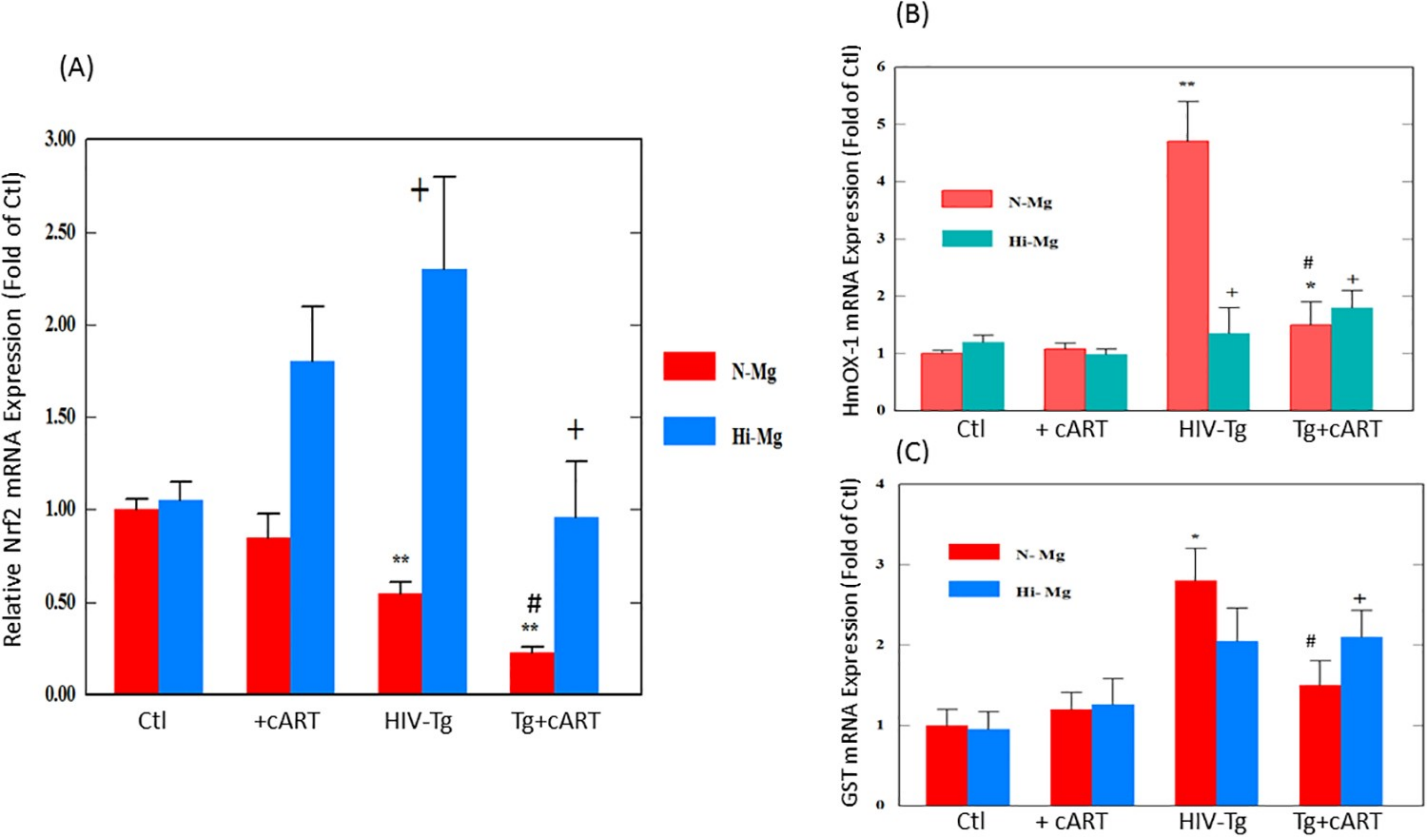
cART suppressed Nfr-2 expression and altered responses of heme oxygenase-1 (HmOX-1) and guthathione-S-transferase (GST) expression in HIV-Tg rats. Control and HIV-Tg rats received cART for 18 months while on normal or high Mg diets; other conditions were as described in Fig 1 legend. Real-time quantitative PCR results for all three hepatic genes were normalized by 18s rRNA. Results are expressed as means of 3–5±SE; *p<0.05, **p<0.01 vs. Ctl, # p<0.05 vs Tg alone, +p<0.05 vs. corresponding normal Mg groups.

Nrf2 is the master transcription factor (Nuclear erythroid-derived factor 2) for the expression of several down-stream antioxidant enzymes [21]. As shown in Fig 3A, cART drug treatment alone did not induce significant change in Nrf2 expression in control rats. However, with HIV-1 expression alone (Tg), the level of Nrf2 mRNA was already decreased by one-half (p<0.01 vs. control); and when combined with cART treatment, the level of Nrf2 expression for the HIV-Tg rats was significantly reduced further (p<0.05 vs. HIV-Tg alone) to about 25% of control. Perhaps the most intriguing observations were that Mg-supplementation not only improved the expression level of Nrf2 in HIV-Tg rats alone, but also substantially raised the Nrf2 expression levels back to 90% of control, even in the presence of cART drug treatment (Fig 3A).

HmOx-1 is one of the downstream antioxidant genes controlled by Nrf2, and is considered protective [22]. Interestingly, Fig 3B shows that HIV transgenic rats alone revealed a much higher expression (>4-fold) of this gene. However, during the cART drug treatment, this upregulation response was blocked (Fig 3B). As represented by Fig 3C, a similar expression pattern was observed for the glutathione S-transferase (GST), which is another Nfr2-governed downstream antioxidant gene [23]. With Mg supplementation, the upregulation of HmOx-1 and GST were reversed in the Tg rats.

We used increases in the circulating level of 8-isoprostane as a surrogated indicator of in vivo oxidative stress [24]. In our published study, we found that this cART treatment promoted an early (6 weeks) elevation of (~3-fold of control) of plasma isoprostane in the Tg rats. At 18 weeks, the cART- treated Tg rats still displayed the highest level of 8-isoprostane, which was about 2.8-fold of control (p<0.01, S1 Table). HIV-Tg alone also displayed a 77% (p<0.05) higher isoprostane level which is consistent with the notion that HIV-1 expression alone experiences certain levels of oxidative stress. As a secondary index of increased oxidative stress, we also measured elevations in RBC GSSG content for the 18-week samples. The normal RBC GSSG content for control rats was about 3% of the total glutathione (GSH+GSSG) and cART treatment increased it slightly to about 4% (S1 Table, p = 0.055). The GSSG content displayed by HIV-1 Tg alone was 4.9% (p<0.01 vs control); and this content was further elevated significantly (p<0.05 vs. Tg alone, <0.01 vs control) to 7.9% by cART treatment. However, with Mg supplementation, all levels of 8-isoprostane, along with the elevated content of RBC GSSG, were significantly suppressed to levels comparable to that of controls (S1 Table).

### Effects of cART and high Mg on expression of selected nitrosative stress-related gene, inducible nitric oxide synthase (iNOS)

**Fig 4:**
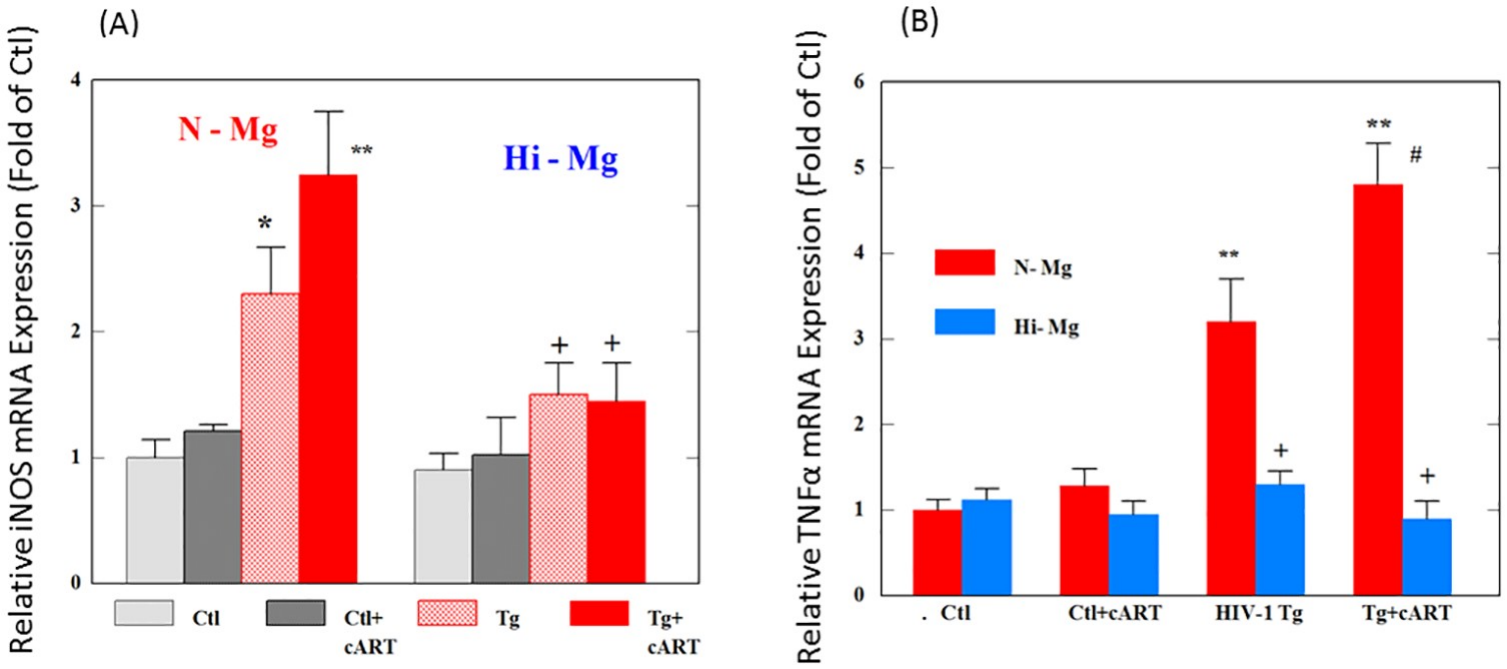
cART up-regulated iNOS and TNF-α in HIV-Tg rats—Blockade by Mg. All conditions were as described in Fig 1 legend. Results are means of 4–5±SE; * p<0.05, **p<0.01 vs. Ctl;, # p<0.05 vs Tg alone, +p<0.05 or 0.025 vs. corresponding N-Mg groups.

We determined the effects of cART, HIV-1 expression and high Mg diet on the mRNA level of iNOS, which is a key gene induced during inflammation and contributes to nitrosative stress [25]. As represented by Fig 4A, HIV-Tg rats alone revealed >2-fold (p<0.05) increase in iNOS expression; and cART treatment of HIV-Tg rats led to a >3-fold higher iNOS expression compared to control (Fig 4A); in separate data, no significant changes in cNOS (constitutive NOS) expression were found in the cART treated Tg rats (96 ± 6% compared to control). Elevated NO reacts rapidly with superoxide to form peroxynitrite (ONOO−), which is a destructive reactive nitrogen free radical species (RNS). 3-NT is a peroxynitrite-mediated protein oxidation product and is often considered a marker of nitrosative stress. The results summarized by S1 Table showed that combined cART drug plus HIV expression caused a significantly higher plasma 3-NT level (>2-fold higher) compared to control. With Mg supplementation, both the elevated iNOS expression and the increased plasma 3-NT level in the Tg ± cART rats were significantly reduced to control levels (Fig 4A, S1 Table).

### Effect of cART, HIV expression and Mg on TNFα expression

TNFα is considered a marker gene of systemic inflammation [26]. Fig 4B indicates that HIV-1 expression alone promoted a significant level of hepatic TNFα expression, and when cART treatment was on board, an even higher level of expression (p<0.05 vs Tg alone) was observed, indicating an enhanced level of inflammation. However, cART alone did not cause a significant change in the control rats. Most importantly, Mg supplementation substantially reduced TNFα expression in both HIV-Tg alone and in cART-treated HIV-Tg rats, to levels that were not significantly different from the control.

## Discussion

Animal models of HIV-1 have played a major role in the investigation of the pathogenesis of HIV and the complex interactions between HIV-1 infection and drugs [27]. The present study demonstrates the differential responses to cART administration between control and HIV-1 Tg rats and the impact of Mg-supplementation on expression of key genes related to oxidative/nitrosative stress, systemic inflammation, and lipogenesis. Although there is no viral replication in the HIV-1Tg rat, viral proteins are continually expressed throughout the animal’s life [28, 29]. This is similar to HIV-1-infected patients receiving cART in which viral replication is substantially suppressed, but viral proteins continue to be expressed and circulated and may have pathological impact [28–31]. Viral transcripts were expressed abundantly in lymph nodes, thymus, liver, kidney, and spleen [28]. Viral proteins (e.g. gp120, Nef [31]) were present in splenic macrophages, T and B cells, and in serum ([28]. Most importantly, HIV-1 Tg rats developed many clinical manifestations of AIDS, including wasting, mild to severe skin lesions, opaque cataracts and varying degrees of kidney and cardiac pathology after 8–9 months of age [28].

A major side effect recognized for most NRTIs is mitochondrial toxicity, and mitochondria-derived oxidative stress is inherently involved [32]. The clinical use of protease inhibitors, especially ritonavir, has been associated with hypertriglyceridemia and hypercholesterolemia [6,7]. SREBP-1 is one of the master transcription factors regulating genes that promote cholesterol and fatty acid biosynthesis [33]. It was reported that HIV-1 expression alone in Tg rats caused disturbances in lipid metabolism resulting in increased accumulation of total cholesterol in liver and hypertriglyceridemia [34]. In agreement with the literature, we observed that HIV-Tg alone displayed a 2.5-fold higher expression of SREBP-1; furthermore, cART treatment led to a 4.3-fold upregulation (p<0.05 vs. Tg alone) of the SREBP-1 gene in the HIV-Tg rats, but not in the control background strain. This finding suggests a synergistic enhancement of SREBP-1 expression in the presence of both cART and HIV-1 expression. Indeed, this enhanced up-regulation was also associated with significant elevations in both total plasma triglyceride and cholesterol levels in the cART-treated Tg rats. Importantly, the enhanced expression of SREBP-1 was substantially suppressed by Mg-supplementation (Fig 1), which resulted in the lowering of circulating cholesterol and triglyceride levels in Tg + cART rats receiving Mg supplementation (Fig 2B).

Nrf2 downregulation has been reported in certain tissues (lung alveolar epithelium [35], whole brain and liver [36] from Tg rats suggesting a compromised antioxidant defense capacity. Indeed, the hepatic Nrf2 gene expression was down-regulated about 50% in the HIV-1 Tg rats alone, and more strikingly, treatment with cART down-regulated this master defensive transcription factor to about 25% of control. By assessing the downstream genes regulated by Nrf2, we were surprised to see that both HmOx-1 and GST expressions were significantly elevated (Fig 3B and 3C) in the Tg rat alone. It was interpreted that even at a 50% level of expression, Nrf2 was still sufficiently active to promote the upregulation of these genes as a protective response against the heightened oxidative stress. However, when the Nrf2 expression was only 25% of control, such compensatory responses were dramatically suppressed by the cART treatment. The prostaglandin-like compound 8-isoprostane forms in vivo from the free radical- catalyzed peroxidation of essential fatty acids (primarily arachidonic acid) without the direct action of cyclooxygenase (COX) enzymes; this compound is regarded as a gold-standard marker of lipid peroxidation in both animal and human models of oxidative stress [37]. Moderate but significant elevations of 8-isoprostane level were observed in HIV-Tg rats on week 6 [8], and here on week 18 (S1 Table), that is consistent with the idea that HIV-1 expression alone exerted a substantial level of oxidative stress [24]. Moreover, the addition of cART treatment to an already compromised antioxidant defense in the Tg animals, contributed to the much higher levels of this lipid peroxidation marker throughout the entire duration (6–18 weeks) of the cART treatment ([8] & S1 Table). In view of the known antioxidant and anti-calcium properties of Mg, it is not surprising that Mg-supplementation suppressed the formation of 8-isoprostane (and GSSG) products. However, the finding that Mg supplementation actually exerted its effects up-stream at the Nrf2 genomic expression level was unexpected. Down-regulation of Nrf2 in both Tg alone and cART–treated Tg rats was attenuated by Mg-supplementation, and alterations in expressions of HmOx-1 and GST were also reversed by Mg.

Both HIV-1 proteins (gp 120 and Tat [38,39]), and ART agents, especially HIV-protease inhibitors [40], were reported to promote endoplasmic reticulum (ER) stress. In vitro studies showed that the HIV-1 proteins, gp 120 or Tat, could directly down-regulate Nrf2 mRNA expression in rat alveolar macrophages [41]. One plausible explanation for the synergy/additive effects of HIV-1 expression plus cART is: increased ER stress might serve as the common oxidative event that promotes both up-regulation of the lipogenic genes (e.g. SREBP-1) and down-regulation of Nrf2. Since HIV-1 proteins and cART treatment can separately enhance ER stress [38–40], in combination, the much heightened and chronic levels of ER stress would be experienced by the Tg animals leading to enhanced genomic changes. The protective effects of Mg-supplementation against altered gene expression might be explained by its effective anti-ROS/anti-nitrosative properties which suppress the heightened oxidative ER stress induced by combined HIV-1 plus cART treatment.

This study also demonstrated that the cART exacerbated nitrosative stress in Tg rats likely due to enhanced expression of iNOS which generates a higher level of NO, and peroxynitrite formation; this was confirmed by the elevated plasma 3-NT level ([8], S1 Table). The expression of iNOS which was upregulated 2-fold in the Tg + cART rats, was also normalized by Mg-supplementation, and this was accompanied by the lowering of plasma 3-NT (S1 Table). In our published results [8], we observed prominent increases in 3-NT-immuno-staining in heart tissue of the Tg+cART-treated rats, suggesting that nitrosative stress was occurring not only systemically, but at the cardiac tissue level; we also found that the NT-staining intensity (of the Tg+cART rat heart) was significantly reduced by Mg-supplementation [8] The overall findings support the notion that Mg supplementation’s anti-peroxidative/anti-nitrosative intervention was effected at the initial hepatic genomic level. HIV-infection alone can stimulate hepatic tumor necrosis factor alpha (TNFα) gene upregulation, due to the enhanced capacity of HIV-specific CD8+ T cells to survive and proliferate; this can lead to an increased production of cytokines and cytotoxic molecules in response to cognate antigen ([42]. Indeed, we confirmed the upregulation of TNFα in HIV-Tg alone, and cART administration further elevated the upregulation, supporting the notion of that cART exacerbated systemic inflammation. Since both iNOS and TNFα were simultaneously up-regulated, the enhanced conversion of the resident macrophages (Kuffer cells) from M2 to M1 stage (possibly by HIV Nef protein) which is pro-inflammatory [43], may be involved.

In conclusion, Mg supplementation can attenuate hyperlipidemia and oxidative/nitrosative/ inflammatory stresses caused by cART in the HIV-1-Tg rats, not only at the biochemical level, but also at the key selected transcriptome level in this model. We submit that these results strongly support a role for Mg supplementation as an adjuvant therapy to suppress abnormal metabolic and oxidative/nitrosative transcription changes potentially caused by cART toxicity in HIV patients.

## Supporting information

S1 Table cART treatment for 18 weeks on plasma oxidative/nitrosative stress indices in control and HIV-Tg rats receiving normal Mg or high Mg diets. (DOCX)

S2 Table Relevant PCR, Lipids data sets for Figs 1–4, and additional oxidative-nitrosative stress indices. (PDF)
