# Supplementary material for: Mg-supplementation attenuated lipogenic and oxidative/nitrosative gene expression caused by Combination Antiretroviral Therapy (cART) in HIV-1-transgenic rats
Source: PLoS One. 2019 Jan 22;14(1):e0210107. doi: 10.1371/journal.pone.0210107 (PMC6342322; doi:10.1371/journal.pone.0210107)
Supplement: S1 Table — (DOCX) [file pone.0210107.s001.docx]

**S1-Table.**

cART treatment for 18 weeks on plasma oxidative/nitrosative stress indices in control and HIV-Tg rats receiving Normal Mg or High Mg diets.

______________________________________________________

**Rat Groups 8-Isoprostane RBC-GSSG 3-Nitrotyrosine** b

(pg/ml) (% GSH + GSSG) (ng/ml Equivalents)

Normal Mg

A-1 (Ctl) 52±5 3.1±0.3 1.26±0.35

A-2 (+cART) 70±6* 4.0±0.25 ^a^ 1.6±0.45

A-3 (Tg) 92±11* 4.9±0.3** 1.8±0.38

A-4 (Tg+cART) 149±18** # 7.9±0.6** # 3.8±0.6** #

High Mg

B-1 (Ctl) 50±6 2.6±0.4 1.0±0.2

B-2 (+cART) 59±7 3.5±0.7 1.2±0.2

B-3 (Tg) 78±7^+^ 3.9±0.4^+^ 1.25±0.1

B-4 (Tg+cART) 82±8**^++^** 4.7±0.6**^++^** 1.3±0.3**^++^**

**__________________________________________________________________________**

Data were based on an average of 4-5 ± SEM; * p<0.05, ** p<0.01 vs A-1 Controls; + p<0.05 vs A-3, ++ p<0.01 vs A-4; # p< 0.05 vs A-3 (Tg); ^a^ p= 0.055 vs A-1(Ctl) Mg diet and drug dose information are in *Material and Methods*. b: plasma 3-nitrotyrosine data were derived from [8].
