# Supplementary material for: Mg-supplementation attenuated lipogenic and oxidative/nitrosative gene expression caused by Combination Antiretroviral Therapy (cART) in HIV-1-transgenic rats
Source: PLoS One. 2019 Jan 22;14(1):e0210107. doi: 10.1371/journal.pone.0210107 (PMC6342322; doi:10.1371/journal.pone.0210107)
Supplement: S2 Table — (PDF) [file pone.0210107.s002.pdf]

S2-Table: Individual data sets for Fig 1-4 and Oxidative/nitrosative stress indices

Fig 1: Individual PCR data for SREBP1 expression (Relative to Control –normal Mg)

Normal Mg diet				High Mg diet			
Ctl	+cART	Tg	Tg+cART	Ctl	+cART	Tg	Tg+cART
1.21	1.65	4.0	6.2	1.4	1.29	1.2	1.3
1.18	1.52	2.6	5.1	1.2	1.16	1.0	0.9
0.85	1.25	2.2	3.9	0.96	1.05	0.6	0.5
0.76	1.18	2.0	3.7	0.8	.79	0.4	0.3
	1.07	1.8	2.8		.76		0.2

Fig 2 Individual lipid data for Triglyceride and Cholesterol levels (mg/dL)

Normal Mg diet **Triglyceride**				High Mg diet **Triglyceride**			
Ctl	+cART	Tg	Tg+cART	Ctl	+cART	Tg	Tg+cART
72	82	89	104	71	81	81	88
65	76	88	99	69	72	75	69
59	64	71	92	51	59	68	65
52	60	79	85	49	61	61	75
	58	70	82		56	55	54

Normal Mg diet **Cholersterol**				High Mg diet **Cholesterol**			
Ctl	+cART	Tg	Tg+cART	Ctl	+cART	Tg	Tg+cART
79	95	94	119	82	94	88	90
76	88	89	116	79	88	77	93
61	71	83	106	71	74	68	72
59	63	70	98	61	60	61	69
		68	95	58	57	53	

Fig 3: (A) PCR data for Nrf2 expression (Relative to Control-normal Mg)

Normal Mg diet				High Mg diet			
Ctl	+cART	Tg	Tg+cART	Ctl	+cART	Tg	Tg+cART
1.1	1.16	0.67	0.3	1.28	2.4	3.8	1.7
1.07	0.98	0.63	0.27	1.16	2.2	2.9	1.3
0.95	0.82	0.45	0.19	0.92	1.8	2.2	0.9
0.88	0.7	0.41	0.16	0.86	1.6	1.4	0.5
	0.6				1.3	1.2	0.4

(B) PCR data for HmOX-1 expression (Relative to Control-normal Mg)

Normal Mg diet				High Mg diet			
Ctl	+cART	Tg	Tg+cART	Ctl	+cART	Tg	Tg+cART
1	1.1	5.1	2.1	1.45	1.01	0.8	1.4
1.13	0.8	4.8	1.1	1.35	0.72	1.4	1.6
0.87	1.33	3.7	1.4	1.2	1.2	1.9	2.6
1.0	1.1	5.3		0.9	1.		2.0

(C) PCR data for GST expression (Relative to Control-normal Mg)

Normal Mg diet				High Mg diet			
Ctl	+cART	Tg	Tg+cART	Ctl	+cART	Tg	Tg+cART
1.3	1.0	3.35	0.8	1.25	1.855	2.0	2.9
0.8	1.1	2.2	2.0	0.9	1.2	1.45	2.5
1.0	1.7	3.0	0.9	0.70	0.85	2.7	2.0
0.9			1.7				1.1

Fig 4: PCR data for iNOS and TNF-alpha expression (Relative to Control-normal Mg)

Normal Mg diet (**iNOS**)

Ctl	+cART	Tg	Tg+cART
1.29	1.35	3.2	4.3
1.15	1.27	2.7	3.5
0.82	1.13	1.9	3.1
0.72	1.1	1.8	2.2
		2.2	

High Mg diet (**iNOS)**

Ctl	+cART	Tg	Tg+cART
1.25	1.4	2.0	2.1
1.13	1.33	1.8	1.75
0.87	0.86	1.2	1.1
0.67	0.5	0.9	1.05

Normal Mg diet (**TNF-alpha**)

Ctl	+cART	Tg	Tg+cART
1.3	1.85	3.9	5.8
1.15	1.2	3.7	5.3
0.95	1.07	2.9	3.7
0.8	0.98	2.2	4.3
0.75			

High Mg diet (**TNF-alpha**)

Ctl	+cART	Tg	Tg+cART
1.39	1.28	1.6	1.3
1.29	1.1	1.0	1.2
1.3	0.8	1.5	0.67
0.9	0.65	1.1	0.5
0.72			

Data sets for S-Table 1 Oxidative and Nitrosative stress Indices

(A) 8-Isoprostane levels (pg/ml)

Normal Mg diet				High Mg diet			
Ctl	+cART	Tg	Tg+cART	Ctl	+cART	Tg	Tg+cART
67	86	120	174	67	51	102	102
57	77	110	197	60	81	61	91
49	69	90	129	39	75	71	81
41	63	72	150	45	50	88	73
41	55	68	95	40	38	68	62

(B) GSSG/Total Glutathione (%)

Normal Mg diet				High Mg diet			
Ctl	+cART	Tg	Tg+cART	Ctl	+cART	Tg	Tg+cART
3.9	4.5	6	9.8	3.9	4.9	4.8	6.5
3.5	4.3	5.2	8.5	2.9	4.3	4.4	5.5
3.0	3.6	4.1	7.8	2.5	2.9	3.8	4.6
2.7	3.7	4.5	7	1.9	1.8	3.4	3.9
2.3		4.8	6.2	1.7		3.1	3.1

(C) Plasma 3-Nitrotyrosine levels (ng/ml)

Normal Mg diet				High Mg diet			
Ctl	+cART	Tg	Tg+cART	Ctl	+cART	Tg	Tg+cART
1.9	2.8	2.6	5.1	1.3	1.7	1.05	1.9
1.8	1.6	2.2	4.5	1.1	1.4	1.5	1.7
0.75	1.3	1.3	3.1	0.9	1.0	1.02	0.9
0.6	0.7	1.2	2.5	0.7	0.8	1.33	0.7
